# Exploring the causal associations between obesity indicators and male reproductive diseases: new evidence from Mendelian randomization

**DOI:** 10.1186/s12610-024-00242-1

**Published:** 2025-11-11

**Authors:** Huijuan Wei, Haoting Chen, Yifei Lin, Haibin Lu

**Affiliations:** 1 Fujian Maternity and Child Health Hospital College of Clinical Medicine for Obstetrics & Gynecology and Pediatrics, Fujian Medical University, Fuzhou, China; 2 The School of Public Health, Fujian Province, Fujian Medical University, Fuzhou, China; 3 The School of Clinical Medicine, Fujian Province, Fujian Medical University, Fuzhou, China; 4 School of Public Health, Hengyang Medical School, University of South China, Hengyang, China

**Keywords:** Male reproductive diseases, Obesity, Mendelian randomization, Maladies de la reproduction masculine, Obésité, Randomisation mendélienne, Dysfonctionnement testiculaire, Dysfonctionnement érectile

## Abstract

**Background:**

The objective of this study was to investigate potential causal associations between indicators of obesity and male reproductive disorders using Mendelian randomization (MR) analysis.

**Methods:**

Based on summary data from the GWAS, we conducted MR analyses. Univariable MR analysis was performed to estimate the association between three obesity indicators and five male reproductive diseases. Multivariable MR analysis was conducted to account for pleiotropy observed in univariable MR analysis by including a set of covariates.

**Results:**

Univariable MR analysis revealed suggestive associations between waist-to-hip ratio (WHR) and testicular dysfunction (OR = 0.32, 95% Cl: 0.11–0.99, *P*_*IVW*_ = 0.049), body mass index (BMI) and erectile dysfunction (OR = 1.28, 95%CI 1.12–1.45, *P*_*IVW*_ = 1.84 × 10^–4^). Multivariate MR analysis indicated after controlling for potential confounders, waist-to-hip ratio was suggestively associated with the decreased risk of testicular dysfunction (OR = 0.23, 95% Cl: 0.08–0.67, *P*_*IVW*_ = 0.008). Nevertheless, multivariate MR analysis also showed that body mass index was suggestively associated with the increased risk of erectile dysfunction (OR = 1.22, 95% Cl: 1.06–1.40, *P*_*IVW*_ = 0.006). Sensitivity analyses confirmed that these results were reliable.

**Conclusion:**

Our two-sample MR analysis suggests generalized obesity in was suggestively associated with the increased risk of erectile dysfunction, while central obesity obesity is associated with an decreased risk of testicular dysfunction.

**Supplementary Information:**

The online version contains supplementary material available at 10.1186/s12610-024-00242-1.

## Résumé

**Contexte:**

L’objectif de cette étude était d'étudier les associations causales potentielles entre les indicateurs d’obésité et les troubles de la reproduction masculine en utilisant une analyse de randomisation mendélienne (RM).

**Méthodes:**

Sur la base des données sommaires de l’étude d’association pangénomique, nous avons effectué des analyses de RM. Une analyse RM univariée a été réalisée pour estimer l'association entre trois indicateurs d'obésité et cinq maladies reproductives masculines. Une analyse RM multivariable a été réalisée pour tenir compte de la pléiotropie observée dans l'analyse RM univariée en incluant un ensemble de co-variables.

**Résultats:**

L’analyse RM univariée a révélé des associations suggestives entre le rapport taille-hanche (RTH) et le dysfonctionnement testiculaire (OR = 0,32, 95% Cl : 0,11-0,99, *P*_IVW_ = 0,049), l'indice de masse corporelle (IMC) et le dysfonctionnement érectile (OR = 1,28, 95%CI 1,12-1,45, _PIVW_ = 1,^84×10-4^). L'analyse RM multivariée a indiqué qu'après avoir contrôlé les facteurs de confusion potentiels, le rapport taille-hanche était associé de manière suggestive à la diminution du risque de dysfonctionnement testiculaire (OR = 0,23, 95% Cl : 0,08-0,67, *P*_IVW_ = 0,008). Néanmoins, l'analyse RM multivariée a également montré que l'indice de masse corporelle était associé de manière suggestive à l'augmentation du risque de dysfonction érectile (OR = 1,22, 95% Cl : 1,06-1,40, *P*_IVW_ = 0,006). Les analyses de sensibilité ont confirmé la fiabilité de ces résultats.

**Conclusion:**

Notre analyse RM à deux échantillons suggère que l’obésité généralisée est associée de manière suggestive à un risque accru de dysfonction érectile, tandis que l’obésité centrale est associée à une diminution du risque de dysfonction testiculaire.

## Study Importance Questions

### What is already known about this subject?

- ➢ Obesity has been associated with various male reproductive disorders, but whether the associations are causal is uncertain.
- ➢ The associations of obesity with male reproductive disorders have not been thoroughly investigated.

### What are the new findings in your manuscript?

- ➢ After adjusting for genetically predicted smoking and alcoholic drinking, the negative associations of genetically predicted waist-to-hip ratio with the risk of testicular dysfunction did persist and strengthen.
- ➢ Genetically predicted higher levels of BMI were associated with a higher risk of erectile dysfunction.
- ➢ According to genetic predictions, indicators of obesity showed no causally associated with with prostatitis, prostate cancer, or male infertility.

### How might your results change the direction of research or the focus of clinical practice?

- ➢ Reducing generalized obesity could be crucial in preventing erectile dysfunction.

## Introduction

Epidemiologic data indicate a high and increasing global prevalence and incidence of common male reproductive diseases [1–4]. Among these disorders, male infertility has been recognized by the World Health Organization (WHO) as a significant global public health issue. Consequently, male reproductive health has garnered considerable attention due to its susceptibility to various influencing factors, including lifestyle choices, environmental influences, and genetic predispositions. Key associated risk factors include obesity, alcohol consumption, smoking, physical inactivity, diabetes, hypertension, and cardiovascular disease [5]. Among these risk factors, obesity has been identified as a prevalent global risk factor significantly impacting male reproductive health, contributing to decreased sperm count and reproductive dysfunction [6, 7]. The high prevalence of obesity and its observed trends underscore its detrimental effects on male reproductive health, leading to increased attention and concern in recent years.

Currently, most cross-sectional and retrospective studies [8, 9] have demonstrated the association between obesity and a wide range of chronic diseases. However, the relationship between obesity and male reproductive diseases remains controversial, with the exact mechanisms and causality between the two not yet fully elucidated. Moreover, the generalizability of some findings is limited due to small sample sizes and selection biases within the study populations. Additionally, traditional observational studies often fail to adequately control for potential confounding factors and reverse causality.

In this study, we propose utilizing MR analysis to investigate the potential association between indicators of obesity and male reproductive disorders. The MR study design offers the advantage of providing more reliable evidence for inferring causality [10]. Additionally, by considering the influence of two specific risk factors—smoking and alcohol consumption [11]—we have incorporated multivariate MR analyses to mitigate the effects of confounding variables and achieve a more accurate estimation of the association between obesity and male reproductive diseases. The application of univariate and multivariable MR analysis in this study addresses the challenges posed by genetic and environmental confounders inherent in traditional observational studies. Consequently, this research will provide a significant scientific foundation for the development of prevention and intervention strategies aimed at improving male reproductive health and enhancing overall well-being.

## Methods

### Study design

**Fig. 1:**
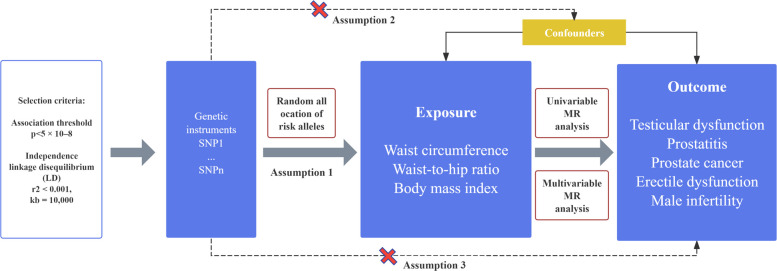
Overview flowchart of assumptions and schematic design. SNPs associated with obesity indicators were used as genetic instruments to study the causal effect of obesity indicators on male reproductive disorders. SNPs associated with obesity indicators were used as genetic instruments to study the causal effect of obesity indicators on male reproductive disorders. Lines with arrows indicate that genetic instruments (SNPs) are associated with exposure and can only influence the outcome through exposure. Dashed lines indicate that the genetic tools (SNPs) are not associated with confounders between the results

MR analysis relies on three fundamental assumptions. First, the genetic variants used as instrumental variables must exhibit a robust association with the exposure under investigation. Second, these selected genetic variants should not be associated with confounding factors. Finally, they should influence the risk of the outcome solely through the relevant risk factors (Fig. 1). In the first step, univariate MR analyses were conducted to estimate the associations between obesity indicators and male reproductive diseases. In the second step, multivariate MR analyses were employed to account for the confounding effects of two key risk factors: smoking and alcohol consumption. This approach enhances the validity of the findings from the univariate MR analyses while systematically minimizing pleiotropy for both traits.

### Data sources

Our study utilized publicly available summary statistics of genetic variants associated with exposure and outcome variables, obtained from large-scale genome-wide association studies (GWAS) conducted on European participants. GWAS is a research methodology to identify genetic variants linked to specific traits or diseases. This approach involves analyzing genome-wide associations between genetic variants, such as single nucleotide polymorphisms (SNPs), and individual phenotypes, including disease states and trait performance. To ensure compliance with ethical guidelines, all original GWAS studies included in this research underwent thorough scrutiny and received approval from the relevant institutional review boards. Additionally, informed consent was obtained from all participants in accordance with the original study protocols. To minimize bias arising from confounding factors, this study employed two-sample Mendelian randomization analyses, using genetic variation as instrumental variables to assess the causal effects of exposures on outcomes.

Fat distribution is closely correlated with endocrine and metabolic processes. Therefore, clinically, obesity is categorized based on the specific areas of fat accumulation into generalized obesity (also known as peripheral or uniform obesity) and central obesity (also referred to as abdominal or visceral obesity). In patients with peripheral obesity, fat primarily accumulates in the limbs and subcutaneous tissue, with a higher prevalence in women. In contrast, central obesity is characterized by the accumulation of visceral fat in the trunk and abdomen, leading to a thicker waist and relatively thinner limbs. This type of obesity is more common in men and is associated with a higher risk of diabetes and other metabolic syndromes [12]. BMI is the most commonly used and recognized standard for measuring obesity, with what we refer to as generalized obesity in everyday language typically indicating peripheral obesity. Waist circumference and waist-to-hip ratio are key initial indicators used to distinguish between peripheral and central obesity. According to WHO standards, a waist circumference of ≥ 90 cm for men and ≥ 85 cm for women, or a waist-to-hip ratio exceeding 1.0, is considered indicative of central obesity [13].

This study builds upon previous research and utilizes publicly available databases from GWAS. It focuses on three exposure variables: central obesity (waist circumference and waist-to-hip ratio), and systemic obesity(BMI). Summary statistics were obtained from the GIANT consortium for waist circumference (*n* = 245,746) [14], waist-to-hip ratio (*n* = 224,452) [14], and body mass index (*n* = 681,275) [15]. Additionally, we investigated five outcome variables: testicular dysfunction, prostatitis, prostate cancer, erectile dysfunction, and male infertility. Testicular dysfunction is a significant male reproductive health issue that encompasses reduced spermatogenesis and sexual dysfunction. These conditions may be associated with various factors, including metabolic syndrome and inflammation [16].

For testicular dysfunction, individual-level GWAS data were obtained from FinnGen, comprising 285 patients with testicular dysfunction and 92,895 controls. The individual-level GWAS data for prostatitis included 1,859 patients with prostatitis and 72,799 controls, also sourced from FinnGen. Summary-level GWAS data for prostate cancer were derived from the PRACTICAL consortium, encompassing 79,148 prostate cancer patients and 61,106 controls [17]. To investigate erectile dysfunction, individual-level GWAS data from a meta-analysis involving three large cohorts were utilized: the UK Biobank (UKBB), the Estonian Genome Center of the University of Tartu (EGCUT), and the Partners HealthCare Biobank (PHB). This analysis included 6,175 patients with erectile dysfunction and 217,630 controls [18]. Cases of erectile dysfunction were primarily identified using ICD-10 codes N48.4 and F52.2 [18]. Regarding male infertility, individual-level GWAS data sourced from FinnGen included 680 patients with male infertility and 217,630 controls. It is important to note that the study population is limited to individuals of European ancestry.

In summary, this study integrates existing research and utilizes publicly available databases from GWAS to explore the associations between central and systemic obesity and conditions such as testicular dysfunction, prostatitis, prostate cancer, erectile dysfunction, and male infertility. The comprehensive information and data sources provided ensure transparency and reliability in the study's methodology.

This study summarizes two potential common risk factors—smoking and alcohol consumption—for the five male reproductive disorders mentioned above, based on previous literature. Summary statistics regarding smoking (*n* = 311,629; controls = 321,173) and alcohol consumption (*n* = 3,353,394) were obtained from the GSCAN consortium [19]. Table 1 presents detailed information about the data sources utilized in the analyses.

**Table 1:** Information of data sources used in the MR study

Traits	Data sources	Sample size (cases/controls)	Number of SNPs	Ancestry	Reference
Waist circumference	GIANT consortium	245,746	2,547,573	Mixed (76% European)	[14]
Waist-to-hip ratio	GIANT consortium	224,452	2,544,137	Mixed (76% European)	[14]
Body mass index	GIANT consortium	681,275	2,336,260	Mixed (76% European)	[15]
Testicular dysfunction	FinnGen	285/92,895	16,378,751	European	-
Prostatitis	FinnGen	1,859/72,799	16,377,460	European	-
Prostate cancer	PRACTICAL consortium	79,148/61,106	20,346,368	European	[17]
Erectile dysfunction	Meta analysis of three cohort studies	6,175/217,630	9,310,196	European	[18]
Male infertility	FinnGen	680/72,799	16,377,329	European	-
Smoking	GSCAN consortium	311,629/321,173	11,802,365	European	[19]
Alcoholic drinking	GSCAN consortium	335,394	11,887,865	European	[19]

### Selection of genetic instrumental variables

In this study, we selected SNPs associated with the exposure factors of interest from the merged Genome-Wide Association Studies database, adhering to the genomic significance threshold (*P* < 5 × 10^–8^) [20]. To estimate linkage disequilibrium, we utilized genomic data from European populations as referenced in the 1000 Genomes Project. For defining linkage disequilibrium, we applied a threshold of r^2^ ≤ 0.001 and kb ≤ 10,000.

When linkage disequilibrium was identified, we retained the single nucleotide polymorphisms that exhibited the strongest linkage with the exposure variable [21]. For SNPs not present in the GWAS results dataset, we utilized appropriate proxy SNPs as substitutes. We excluded SNPs that were not adequately proxied, as well as palindromic SNPs, to ensure that the SNPs employed were statistically significant and independent of each other as genetic instruments. The remaining statistically significant and independent SNPs were subsequently used as genetic tools for Mendelian randomization analyses.

### Statistical analysis

#### Two-sample Mendelian Randomization Analysis

We performed a two-sample MR analysis to investigate the association between obesity indicators and male reproductive disorders. The primary MR method employed was the inverse variance weighting (IVW) method, a standard approach for summarizing MR data that allows for the direct estimation of causal relationships between study subjects based on pooled data [22]. The Mendelian Randomization Pleiotropy RESidual Sum and Outlier (MR-PRESSO) method was used to detect outliers in the IVW linear regression and to adjust the MR estimates after removing these outliers. Other complementary methods included fixed-effects IVW, weighted median, weighted mode, and simple mode (Fig. 1). The median-based methods require that at least half of the genetic instruments in the pooled data be valid to obtain consistent effect estimates. These methods include unweighted median (simple median), weighted median, and penalized weighted median estimates. The weighted median combines both the weighted estimate and the median to more accurately assess the effect of each genetic variant, accounting for varying causal effect weights [23, 24]. Moreover, if the IVW method yields statistically significant results while other methods do not, the odds ratios (ORs) derived from the alternative methods must align with the direction of the IVW results. If they do not, their statistical significance is questionable. Furthermore, multivariate MR, an extension of univariate MR, enables the joint detection of causal effects from multiple risk factors [25]. This approach has been used to adjust for differences in the genetic prediction of obesity indicators, smoking, and alcohol consumption [26].

To avoid increasing the risk of Type I errors when performing multiple statistical tests, we applied the Bonferroni correction to adjust the significance thresholds. Consequently, we identified strong evidence at a significance level of *p* < 0.003 (three exposures and five outcomes) and suggestive evidence at a significance level of 0.003 ≤ *p* < 0.05 in the univariable MR analysis.

### Sensitivity analyses

Sensitivity analyses were conducted to investigate the association between obesity and male reproductive conditions. Several statistical methods were employed, including Cochrane's Q statistic, the MR-Egger intercept, and the Mendelian Randomization Pleiotropy Residual Sum and Outlier (MR-PRESSO), to assess potential heterogeneity and horizontal pleiotropy in the primary analyses.

In this study, Cochrane's Q statistic was utilized to evaluate heterogeneity, with a significance level of *p* < 0.05 indicating the presence of heterogeneity. The MR-Egger intercept and MR-PRESSO analyses were employed to assess horizontal pleiotropy. In MR-Egger regression, the intercept term served as a valid indicator of directional horizontal pleiotropy, with *p* < 0.05 denoting its presence [27]. The MR-PRESSO analysis consisted of three main steps [28]. Firstly, a horizontal pleiotropy test was conducted to identify potential pleiotropic effects. Subsequently, the multivariate correction was applied to remove outliers caused by genetic variants identified as exhibiting horizontal pleiotropy. Finally, a comparison was performed between the data before and after correction to examine any differences in causal associations.

All statistical analyses were conducted using R version 4.2.2 (R Foundation for Statistical Computing, Vienna, Austria). MR analyses were performed using the TwoSampleMR, MR-PRESSO, and MRInstruments R packages.

## Result

### Univariable MR analysis of obesity and male reproductive diseases

We conducted a two-way, two-sample MR study to investigate the relationship between obesity indicators and male reproductive diseases. The F-statistics for waist circumference, waist-to-hip ratio, and BMI were all greater than 10 (STable 1–15).

**Fig. 2:**
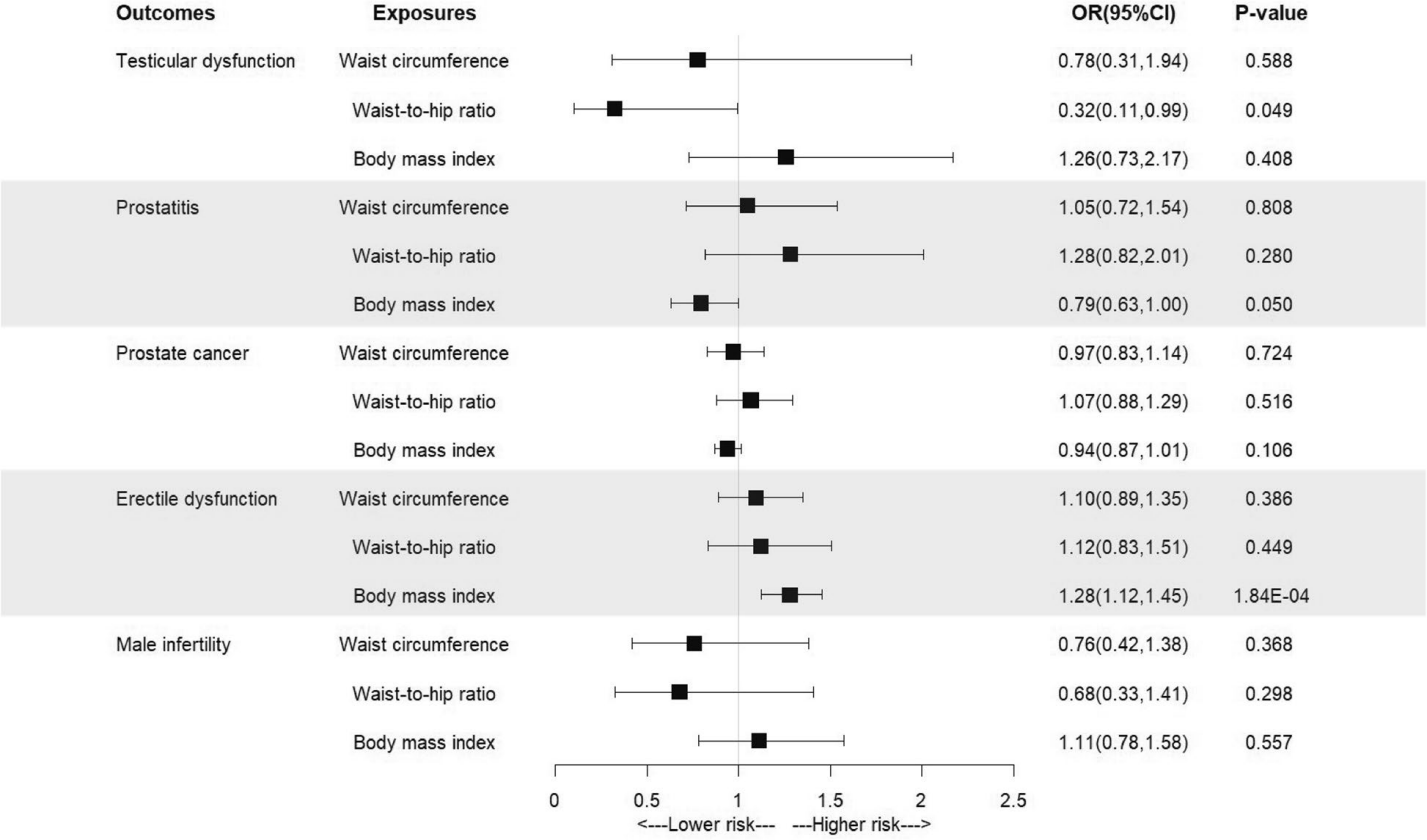
Univariable MR analysis of obesity and male reproductive disorders

As shown in Fig. 2, suggestive evidence was found for the association between waist-to-hip ratio and testicular dysfunction (OR [95% CI] = 0.32 [0.11–0.99], *P* = 0.049). However, we found no evidence of a correlation between waist circumference or BMI and testicular dysfunction. Furthermore, the results indicated strong evidence for the association between body mass index and erectile dysfunction (OR [95% CI] = 1.28 [1.12–1.45], *P* = 1.84 × 10⁻^4^), while no significant correlation was observed between waist circumference or waist-to-hip ratio and erectile dysfunction. Additionally, no evidence of correlation was found between waist circumference, waist-to-hip ratio, body mass index, and prostatitis, prostate cancer, or male infertility.

Heterogeneity may exist among the instrumental variables for some exposures (Table 2). Consequently, we employed the random-effects inverse-variance weighted Mendelian randomization (IVW-MR) method as the primary analytical approach. The MR-Egger intercept and MR-PRESSO analyses indicated a potential presence of horizontal pleiotropy in the instrumental variables related to waist-to-hip ratio (MR-Egger intercept *P* = 0.042). In contrast, no horizontal pleiotropy was observed for the other exposure factors (Table 2).

**Table 2:** Sensitivity analysis of association between obesity and male reproductive conditions

Outcomes	Exposures	nSNPs	beta	se	*P* value	Cochrane Q test pvalue	MR-Egger intercept pvalue	Global test pvalue	Distortion test pvalue
Testicular dysfunction	Waist circumference	63	-0.254	0.469	0.588	0.857	0.513	0.880	NA
	Waist-to-hip ratio	35	-1.129	0.574	0.049	0.817	0.042	0.837	NA
	Body mass index	485	0.230	0.278	0.408	0.334	0.492	0.400	NA
Prostatitis	Waist circumference	63	0.047	0.195	0.808	0.312	0.468	0.355	NA
	Waist-to-hip ratio	35	0.248	0.230	0.280	0.511	0.507	0.515	NA
	Body mass index	485	-0.231	0.118	0.050	0.012	0.081	< 0.001	0.376
Prostate cancer	Waist circumference	63	-0.028	0.081	0.724	0.000	0.437	< 0.001	0.141
	Waist-to-hip ratio	35	0.064	0.099	0.516	0.000	0.420	< 0.001	0.166
	Body mass index	493	-0.063	0.039	0.106	0.000	0.356	< 0.001	0.889
Erectile dysfunction	Waist circumference	63	0.092	0.106	0.386	0.838	0.685	0.854	NA
	Waist-to-hip ratio	35	0.114	0.151	0.449	0.098	0.302	0.099	NA
	Body mass index	495	0.246	0.066	0.000	0.023	0.327	0.016	0.970
Male infertility	Waist circumference	63	-0.275	0.305	0.368	0.697	0.726	0.707	NA
	Waist-to-hip ratio	35	-0.389	0.373	0.298	0.660	0.425	0.689	NA
	Body mass index	485	0.105	0.178	0.557	0.614	0.899	0.680	NA

### Multivariable MR analysis of obesity and male reproductive diseases

**Fig. 3:**
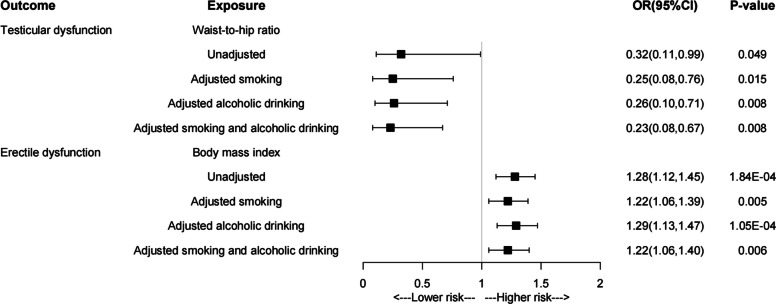
Multivariable MR analysis of obesity and male reproductive disorders

After adjusting for smoking and alcohol consumption, the protective effect of the WHR on testicular dysfunction was enhanced (WHR, 0.23 [0.08–0.67], *P* = 0.008), while the risk associated with erectile dysfunction was attenuated (BMI, 1.22 [1.06–1.40], *P* = 0.006) (Fig. 3). Therefore, our two-sample MR analysis suggests that central obesity is suggestively associated with a decreased risk of testicular dysfunction, whereas generalized obesity is suggestively associated with an increased risk of erectile dysfunction.

## Discussion

### Principal Findings

The present study employed a Mendelian randomization approach to reveal the associations between obesity and male reproductive disorders. Our findings indicate a negative association between central obesity and testicular dysfunction, while generalized obesity is positively associated with erectile dysfunction. These results have significant clinical implications for enhancing our understanding of the relationship between obesity and male reproductive health, as well as for developing effective prevention strategies.

Our findings indicate a protective effect of the waist-to-hip ratio on testicular dysfunction, which is further enhanced after adjusting for smoking and alcohol consumption. Notably, our study is the first to explore the genetic-level association between central obesity and testicular dysfunction. However, the results from existing literature suggest that obesity is a risk factor for male hypogonadism [29, 30], which is inconsistent with our findings. The specific reasons for this discrepancy require further investigation in future studies.

There was a positive association between BMI and erectile dysfunction. However, after adjusting for smoking and alcohol consumption, the impact of BMI on the risk of erectile dysfunction was attenuated. Our findings are consistent with a cross-sectional study that reported a higher risk of erectile dysfunction in obese men (OR [95% CI] = 1.97 [1.25–3.14], *P* = 0.004) [31]. Furthermore, the results of several other studies also support our findings [31–34]. Notably, this study is the first to analyze the association between generalized obesity and erectile dysfunction at the genetic level.

Furthermore, no significant correlation was found between obesity indicators and the other three male reproductive disorders. Parikesit et al. demonstrated that there is currently insufficient evidence to establish an association between obesity and prostatitis, a finding that aligns with the results of our study [35]. Similarly, the conclusion by Adriana C. et al. that BMI is not associated with prostate cancer-specific mortality risk somewhat supports our findings [36]. Additionally, the study by Harrison et al. suggests limited evidence of an association between BMI and the risk of prostate cancer or advanced prostate cancer, further corroborating our results [37]. However, our findings are inconsistent with the conclusions of Nguyen et al., who reported that obesity is a risk factor for male infertility (OR [95% CI] = 1.36 [1.13–1.63]) [38]. We propose that the disparity in findings may arise from confounding factors and reverse causal associations present in previous studies, highlighting the need for further research to provide definitive confirmation. These discrepancies suggest that different indicators of obesity may play varied roles in the pathogenesis of male reproductive disorders.

In conclusion, our study indicates that central obesity positively impacts the prevention of testicular dysfunction, while generalized obesity is associated with an increased risk of erectile dysfunction. Therefore, appropriately increasing the waist-to-hip ratio within a healthy BMI range may be more beneficial for men's reproductive health.

### Strengths and Limitations

This study has several notable advantages. Firstly, it is the first investigation to explore the relationship between obesity and male reproductive diseases from a genetic perspective. Compared to traditional observational studies, our use of an MR design allowed us to minimize residual confounding and avoid reverse causality bias, thereby strengthening the validity of our findings [39]. Furthermore, to enhance the reliability of our results, we implemented a variety of supplementary methods and conducted sensitivity analyses [40].

However, Mendelian randomization study designs have inherent limitations that must be considered. Firstly, it is important to note that the population included in the GWAS database used for our study was predominantly of European descent. Consequently, the generalizability of our findings to other populations may be limited. To enhance the understanding of the association between obesity and male reproductive disorders, we recommend establishing more high-quality, large-scale epidemiological cohorts that encompass diverse populations, similar to the FinnGen project [41]. Including participants from various genetic backgrounds will facilitate the identification and validation of these associations across different populations. Secondly, it is essential to acknowledge the potential presence of pleiotropy, which can introduce bias into the results. However, we employed several strategies to mitigate the impact of pleiotropy bias. These strategies included utilizing the MR-Egger intercept to identify pleiotropy and applying MR-PRESSO analysis to detect outliers in the data. Finally, it is important to recognize that the MR analysis in this study is based on aggregated data and, therefore, does not allow for a detailed exploration of potential differences in associations among various subgroups of the population at the individual level. Lastly, the waist-to-hip ratios in the data source were not accurately recorded within the specified ranges, which may have introduced certain limitations in the results. Therefore, while our study offers new insights, further research and experiments are necessary to investigate individual-level data. Such efforts will contribute to a more comprehensive understanding of the relationship between obesity and male reproductive disorders.

## Conclusion

Our two-sample MR analysis suggests that generalized obesity is associated with an increased risk of erectile dysfunction. Furthermore, larger GWAS databases will be necessary in the future to confirm this relationship and ensure the robustness and reliability of the study's findings.

## Supplementary Information

Supplementary Material 1.

## Data Availability

The datasets generated during and/or analyzed during the current study are available in the Github repository, https://github.com/zoudasheng/MR-BMRandAD.git. Some data generated or analyzed during this study are included in this published article/as supplementary information files.

## Abbreviations

BMI: Body mass index
EGCUTE: Sto-nian Genome Center of the University of Tartu
GWAS: Genome-wide association studies
IVW-MR: Inverse-variance weighted Mendelian randomization
MR: Mendelian randomization
MR-PRESSO: MR-pleiotropy residual sum and outlier
PHB: Partners HealthCare Bio-bank
SNPs: Single nucleotide polymorphisms
UKBB: UK Biobank
WHO: World Health Organization
WHR: Waist-to-hip ratio
